# Hypoxic‐induction of arginase II requires EGF‐mediated EGFR activation in human pulmonary microvascular endothelial cells

**DOI:** 10.14814/phy2.13693

**Published:** 2018-05-20

**Authors:** Caitlyn M. Pool, Yi Jin, Bernadette Chen, Yusen Liu, Leif D. Nelin

**Affiliations:** ^1^ Pulmonary Hypertension Group Center for Perinatal Research Research Institute at Nationwide Children's Hospital Columbus Ohio; ^2^ Department of Pediatrics The Ohio State University Columbus Ohio

**Keywords:** Cellular proliferation, pulmonary endothelial cells, pulmonary hypertension, pulmonary vascular remodeling

## Abstract

We have previously shown that hypoxia‐induced proliferation of human pulmonary microvascular endothelial cells (hPMVEC) depends on arginase II, and that epidermal growth factor receptor (EGFR) is necessary for hypoxic‐induction of arginase II. However, it remains unclear how hypoxia activates EGFR‐mediated signaling in hPMVEC. We hypothesized that hypoxia results in epidermal growth factor (EGF) production and that EGF binds to EGFR to activate the signaling cascade leading to arginase II induction and proliferation in hPMVEC. We found that hypoxia significantly increased the mRNA levels of EGF, EGFR, and arginase in hPMVEC. Hypoxia significantly increased pEGFR(Tyr845) protein levels and an EGF neutralizing antibody prevented the hypoxic induction of pEGFR. Inhibiting EGFR activation prevented hypoxia‐induced arginase II mRNA and protein induction. Treatment of hPMVEC with exogenous EGF resulted in greater levels of arginase II protein both in normoxia and hypoxia. An EGF neutralizing antibody diminished hypoxic induction of arginase II and resulted in fewer viable cells in hPMVEC. Similarly, siRNA against EGF prevented hypoxic induction of arginase II and resulted in fewer viable cells. Finally, conditioned media from hypoxic hPMVEC induced proliferation in human pulmonary artery smooth muscle cells (hPASMC), however, conditioned media from a group of hypoxic hPMVEC in which EGF were knocked down did not promote hPASMC proliferation. These findings demonstrate that hypoxia‐induced arginase II expression and cellular proliferation depend on autocrine EGF production leading to EGFR activation in hPMVEC. We speculate that EGF‐EGFR signaling may be a novel therapeutic target for pulmonary hypertensive disorders associated with hypoxia.

## Introduction

Vasoconstriction and vascular remodeling in the pulmonary circulation are the hallmarks of pulmonary hypertension. There are currently no available therapies specifically targeting the abnormal vascular remodeling that underlies the pathogenesis of pulmonary hypertension (PH). The PH‐associated abnormal vascular remodeling depends on cellular proliferation in the vessel wall (Morrell et al. 2009; Dai et al. 2016). PH is also associated with chronic lung diseases and high altitude, which have a component of hypoxia (Simonneau et al. 2009). We have shown in both human pulmonary microvascular endothelial cells (hPMVEC) and in human pulmonary artery smooth muscle cells (hPASMC) that hypoxia‐induced cellular proliferation depends on arginase II (Chen et al. 2009, 2012; Toby et al. 2010; White et al. 2017; Xue et al. 2017). Arginase II catalyzes the hydrolysis of l‐arginine to l‐ornithine and urea (Nelin et al. 2007). The l‐ornithine produced by arginase II can then be further metabolized to polyamines and proline, which are essential for cellular proliferation (Nelin et al. 2007; Morris 2009). We have previously shown that in hPMVEC the hypoxic induction of arginase II depends on epidermal growth factor receptor (EGFR) activation (Toby et al. 2010). Recently, we reported that EGFR activation leads to the activation of extracellular signal‐regulated kinase (ERK), and this EGFR‐ERK pathway is necessary for hypoxia‐induced arginase II expression (White et al. 2017). Although our data demonstrate a central role for EGFR activation in hypoxic arginase II‐dependent cellular proliferation, the exact mechanism by which EGFR is activated in hypoxic hPMVEC remains unclear. EGFR can be activated by ligand binding, and its preferred ligand is epidermal growth factor (EGF), although there are other ligands which bind EGFR as well (Singh et al. 2016). When activated by ligand binding, two EGFR moieties dimerize resulting in transphosphorylation that enables interactions with cell signaling molecules (Fuller et al. 2008). We have previously shown that EGF treatment of bovine pulmonary arterial endothelial cells resulted in EGFR phosphorylation and activation (Nelin et al. 2005). Therefore, we hypothesized that hypoxia results in EGF production and that the EGF produced leads to the hypoxic induction of arginase II and proliferation in hPMVEC via EGFR activation. We studied hPMVEC using qPCR, Western blotting, and an assay for viable cell numbers. An antibody that binds with EGF to prevent EGF binding to surface receptors was used, and we called this an EGF neutralizing antibody. To prevent EGF production by cells we utilized EGF siRNA. An antibody that binds with EGFR to prevent ligand binding was used, and we called this an EGFR blocking antibody. Finally, we performed cell proliferation studies in hPASMC using conditioned media from hPMVEC.

## Methods

### Human pulmonary microvascular endothelial cell culture

Human pulmonary microvascular endothelial cell were cultured as previously described (Toby et al. 2010; White et al. 2017). Briefly, hPMVEC were purchased from Lonza (catalog number cc‐2527, lot # 0000366560, Allendale, NJ) and cultured in endothelial cell media (ECM; ScienCell, Carlsbad, CA) supplemented with an ECM kit containing FBS, ECGS, and a penicillin/streptomycin solution (ScienCell). hPMVEC between passages 4 and 10 were used for these studies. On the day of study, the hPMVEC were washed with 2 mL of Dulbecco's phosphate‐buffered saline (DPBS; Corning Life Sciences, Tewksbury, MA). Then 1 mL of ECM was placed on the hPMVEC and the hPMVEC were returned to the incubator at 37°C in either 5% CO_2_, balance air (normoxia) or 5% CO_2_, 1% O_2_, balance N_2_ (hypoxia) for either 24 or 48 h. Depending on the experimental protocol, 50 ng/mL EGF (BioVision, Milpitas, CA), 2 *μ*mol/L AG1478 (A.G. Scientific, San Diego, CA), 20 *μ*g/mL EGFR blocking antibody (EMD Millipore, Temecula, CA), and/or either 0.2, 0.5, or 1.0 *μ*g/mL EGF neutralizing antibody (R&D Systems, Minneapolis, MN) were added to the medium. The respective vehicles were added to the ECM medium as controls. All treatments were added at time 0, the start of the exposure to either normoxia or hypoxia, of the given experiment. The control for the antibody experiments was isotype IgG. The cells were harvested for protein extraction or RNA isolation. For the siRNA experiments, hPMVEC were treated with vehicle, a scramble siRNA, or siRNA targeting EGF (SMARTpool^®^ siGENOME EGF siRNA, catalog number M‐011650‐01‐0005, Dharmacon, Lafayette, CO) using Dharmafect (Dharmacon) transfection reagent as previously described (Toby et al. 2010; White et al. 2017). After 24 h, the hPMVEC were washed and allowed to recover in normoxia for 48 h. The hPMVEC were then used in the experimental protocols.

### Human pulmonary artery smooth muscle cells

hPASMC (Lonza, catalog #CL‐2581, lot #7F3558) were cultured as previously described (Chen et al. 2009, 2012; White et al. 2017). Briefly, hPASMC were grown in 5% CO_2_ at 37°C in smooth muscle growth media (SmGM‐2; Lonza), which include smooth muscle basal medium (Lonza), 5% FBS, 0.5 ng/mL human recombinant EGF, 2 ng/mL human recombinant fibroblast growth factor, 5 *μ*g/mL insulin, and 50 *μ*g/mL gentamicin. The hPASMC were used in experiments between the fifth and eighth passages, throughout which no changes in cell morphology were noted.

### Protein isolation

Protein was isolated as previously described (Toby et al. 2010; Setty et al. 2017; Xue et al. 2017). Briefly, hPMVEC were washed with DPBS and 50 *μ*L lysis solution (20 mmol/L HEPES, pH 7.4; 50 mmol/L *β*‐glycerophosphate, 2 mmol/L EGTA, 1 mmol/L DTT, 10 mmol/L NaF, 1 mmol/L Na_3_VO_4_, 1% Triton X‐100, 10% glycerol) was added. Thirty minutes before use, the following protease inhibitors were added to each milliliter of lysis solution: 1 *μ*g aprotinin, 1 *μ*g leupeptin, 1 *μ*g pepstatin A, and 1 *μ*g phenylmethylsulfonyl fluoride. The hPMVEC were scraped and placed in sterile centrifuge tubes on ice for 30 min. The samples were centrifuged at 20,000*g* for 15 min at 4°C. The supernatant was stored at −80°C for subsequent Western blot analysis. Total protein concentration was determined by the Bradford method using a commercially available assay kit (BioRad, Hercules, CA).

### RNA isolation

RNA was isolated as previously described (Nelin et al. 2005; Toby et al. 2010). Briefly, hPMVEC were washed with DPBS. Then trizol (Invitrogen, Carlsbad, CA) was added to the cells and transferred to sterile centrifuge tubes to be incubated for 5 min at room temperature. Chloroform was added, and the tubes were shaken for 30 sec and then incubated at room temperature for 3 min. The mixture was centrifuged at 12,000*g* for 15 min at 4°C. The supernatant was transferred to a fresh tube. Isopropyl alcohol was added, and the mixture was incubated at room temperature for 10 min and then centrifuged at 12,000*g* for 15 min at 4°C. The supernatant was discarded, and the pellet was washed with 75% ethanol and centrifuged at 7500*g* for 5 min at 4°C. The supernatant was discarded and the pellet was partially dried, dissolved in RNase‐free water, and stored at −80°C.

### Western blotting

Cell lysates were assayed for arginase II and *β*‐actin using Western blot analysis as previously described (Nelin et al. 2005; Chen et al. 2009, 2012; White et al. 2017; Xue et al. 2017). Aliquots of cell lysate were diluted with appropriate amounts of 10x NuPAGE reducing agent, 4x NuPAGE LDS sample buffer, and deionized water. The samples were then heated to 80°C for 10 min, and then separated by electrophoresis using NuPAGE gels (Invitrogen). The proteins were transferred to polyvinylidene difluoride membranes and blocked in Tris‐buffered saline with 0.1% Tween (TBS‐T) containing 10% skim milk for 1 h. The membranes were then washed once with TBS‐T and incubated with a rabbit primary antibody against arginase II (1:500; Santa Cruz Biotechnology, Dallas, TX, catalog # sc‐20151, lot # A2512) for 1 h. The membranes were washed three times with TBS‐T and incubated with goat horseradish peroxidase (HRP) conjugated anti‐rabbit IgG secondary antibody (1:15,000; Bio‐Rad) for 1 h. Then the membranes were washed three times with TBS‐T. The bands for arginase II were visualized using chemiluminescence (Amersham ECL, Piscataway, NJ) and quantified using densitometry (Total Lab, Newcastle upon Tyne, UK). To control for protein loading, the blots were stripped using a stripping buffer (62.5 mmol/L Tris‐HCl pH 6.8, 2% SDS, 100 mmol/L *β*‐mercaptoethanol) and reprobed for *β*‐actin using a monoclonal antibody (1:10,000; Sigma, St Louis, MO, catalog # A1978‐200UL, control # 010M4816).

### Immuonoprecipitation

hPMVECs were harvested with denaturing cell lysis buffer containing 50 mmol/L Tris (pH7.5), 0.5% SDS, and 70 mmol/L *β*‐mercaptoethanol. The cell lysate was boiled, cooled down, and then diluted 5x with ice‐cold cell lysis buffer (50 mmol/L Tris (pH 7.5), 1% Triton X‐100, and 14 mmol/L *β*‐mercaptoethanol). The samples were centrifuged at 20,000*g* for 15 min at 4°C and the supernatant was removed and stored at −80°C. Some of the cell lysate (referred to as whole‐cell lysate or WCL) was used for Western blotting for pEGFR(Tyr845) (Cell Signaling Technology, Danvers, MA, catalog #2231 lot # 1) or total EGFR (1:1000 Abcam, Cambridge, MA, catalog #ab131498). The cells lysate was then mixed with pTyr antibody (4G10; Millipore, Billerica, MA, catalog # 05‐321, lot # DAM1411323) and gently rocked for 1 h at 4°C. Protein G Plus‐agarose (Santa Cruz Biotechnology) was then added to the lysate and incubated at 4°C overnight. The mix was centrifuged at 660*g* for 5 min at 4°C. The beads were then washed 5 times with the cell lysis buffer, resuspended with the electrophoresis sample buffer, boiled, and loaded on NuPAGE gels for electrophoresis and Western blot analysis. The membrane was probed with a pEGFR(Y845) antibody (Cell Signaling Technology, Danvers, MA, catalog #2231, lot# 1).

### Real‐time PCR

Real‐time PCR for EGF, EGFR, and arginase II was performed as previously described (Nelin et al. 2005; Toby et al. 2010; White et al. 2017). DNase treatment was performed on all samples using RNase‐free DNase (Super Array, SA Biosciences, Frederick, MD) followed by reverse transcription (Promega Corp., Madison,WI) and then analysis of cDNA by real‐time PCR using Absolute Blue qPCR SYBR Green (Thermo Fisher Scientific, Walthem, MA). Primers were ordered from IDT (Integrated DNA Technologies Coralville, IA) using the following sequences for human EGFR‐forward primer: 5′ TTTGCTGATTCAGGCTTGG 3′; reverse primer: 5′ AGAAAACTGACCATGTTGCTTG 3′. Primers were ordered from Invitrogen using the following sequences for human EGF‐forward primer: 5′ GGGAATGGTTTATGCCCTAGAT 3′; reverse primer: 5′ CGCTGGGAACCATCCATATT 3′ and for human arginase II‐forward primer: 5′ TTAGCAGAGCTGTGTCAGATGGCT 3′; reverse primer: 5′ GGGCATCAACCCAGACAACACAAA 3′. 18S was amplified using the forward primer (5′ CCAGAGCGAAAGCATTTGCCAAGA 3′) and the reverse primer (5′ TCGGCATCGTTTATGGTCGGAACT 3′). For each reaction, negative controls containing reaction mixture and primers without cDNA were performed to verify that primers and reaction mixtures were free of template contamination. Relative EGFR, EGF, and arginase II mRNA amounts were normalized to 18S expression using the ∆∆CT method. All samples were analyzed in duplicate. Data are shown as fold change relative to normoxia‐exposed hPMVEC controls at each respective time point.

### Trypan blue exclusion for determining viable cell numbers

Viable cell numbers in hPMVEC and hPASMC were determined as previously described (Chen et al. 2009; Toby et al. 2010; Setty et al. 2017; White et al. 2017). For hPMVEC 5 × 10^4^ cells were plated in each well of a six‐well plate, and for hPASMC 1 × 10^4^ cells were plated in each well of a six‐well plate. The appropriate treatments were included in the media and the cells were placed in either hypoxia or normoxia for a period of 48 h for hPMVEC and 120 h for hPASMC. At the end of the experimental protocol the cells were washed twice with DPBS. After the final wash, 1 mL of trypsin was added to each well. The plates were incubated for 3 min followed by the addition of 2 mL trypsin neutralizing solution. The cells from each well were placed in 15 mL conical tubes. The cells were centrifuged for 5 min at 1220*g* at 4°C. The supernatant was discarded and the cells were resuspended in 1 mL of ECM. The cells were then mixed 1:1 with trypan blue solution and viable cells were counted using a hemocytometer.

### Statistical analysis

Values are expressed as the means ± SE. One‐way analysis of variance (ANOVA) was used to compare the data between groups. Significant differences were identified using a Neuman–Keuls post hoc test (SigmaStat 12.5, Jandel Scientific, Carlsbad, CA). Differences were considered significant when *P* < 0.05.

## Results

### Hypoxia increased arginase II, EGF, and EGFR mRNA expression

**Figure 1:**
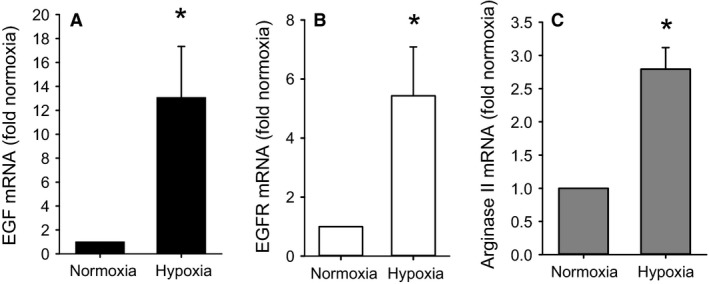
Hypoxia increased expression of EGF, EGFR, and arginase II. hPMVEC were incubated for 24 h in either normoxia or hypoxia and RNA isolated for qPCR for EGF (A), EGFR (B) and arginase II (C). *n* = 3 for each group. Gene expression levels were expressed as fold relative to those in normoxia. * hypoxia different from normoxia, *P* < 0.05.

To determine the role of hypoxia on EGF and arginase II mRNA expression and to corroborate our previous findings regarding EGFR mRNA expression (Toby et al. 2010), hPMVEC were incubated in either normoxia or hypoxia for 24 h and the RNA was harvested for qPCR. Incubating hPMVEC in hypoxia resulted in ~13‐fold greater EGF mRNA levels than in hPMVEC incubated in normoxia (Fig. 1A). As we have shown previously (Toby et al. 2010), incubating hPVMEC in hypoxia resulted in significantly greater EGFR mRNA levels than in normoxic hPMVEC (Fig. 1B). Consistent with our previous findings regarding arginase II protein levels (Toby et al. 2010; White et al. 2017) hPMVEC incubated in hypoxia had significantly greater mRNA levels of arginase II than did hPVMEC incubated in normoxia (Fig. 1C).

### Inhibiting EGFR signaling prevented hypoxia‐induced arginase II expression

**Figure 2:**
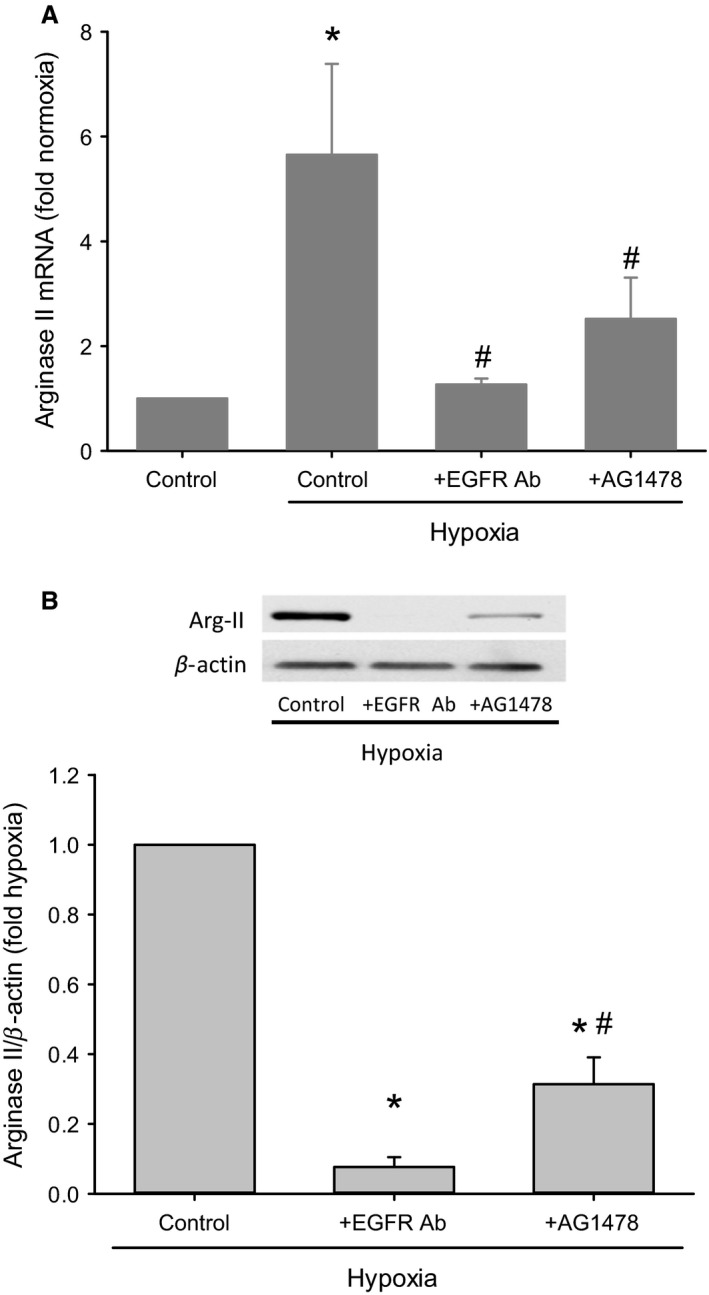
Inhibiting ligand binding to EGFR or the tyrosine kinase activity of EGFR prevented hypoxia‐induced arginase II expression. (A) hPMVEC were treated with either 10 *μ*mol/L AG1478 (+AG1478) or 20 *μ*g/mL EGFR blocking antibody (+EGFR Ab) and then incubated for 24 h in hypoxia, non‐treated cells were incubated in either normoxia or hypoxia for 24 h, and RNA isolated for qPCR for arginase II (*n* = 3 for each group). * hypoxia different from normoxia, *P* < 0.05. # hypoxia + treatment different from nontreated hypoxia, *P* < 0.05. (B) hPMVEC were not treated (control) or treated with either 10 *μ*mol/L AG1478 or 20 *μ*g/mL EGFR blocking antibody and then incubated for 24 h in hypoxia. Protein was isolated for arginase II immunoblotting; *n* = 3 for each group. Typical Western blot for arginase II and *β*‐actin is shown above and the densitometry results for arginase II normalized to *β*‐actin are shown below as fold change from control. * different from control, *P* < 0.001; # +EGFR Ab different from +AG1478, *P* < 0.05.

To investigate the effect of EGFR signaling on arginase II mRNA and protein expression, EGFR blocking antibody or AG1478 were utilized. hPMVEC were not treated (control) or treated with either 10 *μ*mol/L AG1478 or 20 *μ*g/mL EGFR blocking antibody and then incubated for 24 h in hypoxia. A group of nontreated hPMVEC were incubated in room air for 24 h. RNA was isolated for qPCR for arginase II. Treatment with the EGFR blocking antibody prevented the hypoxia‐induced increase in arginase II mRNA levels (Fig. 2A). Treatment with AG1478 significantly attenuated the hypoxia‐induced increase in arginase II mRNA levels (Fig. 2A). In a second set of studies, hPMVEC were treated with either 10 *μ*mol/L AG1478 or 20 *μ*g/mL EGFR blocking antibody and then incubated for 24 h in hypoxia. Protein was isolated for arginase II immunoblotting. Treatment of hPMVEC with EGFR blocking antibody prevented hypoxia‐induced arginase II protein expression (Fig. 2B). Similarly, treatment of hPVMEC with AG1478 significantly attenuated the hypoxia‐induced arginase II protein levels. (Fig. 2B).

### EGF increased arginase II protein expression in hPMVEC

**Figure 3:**
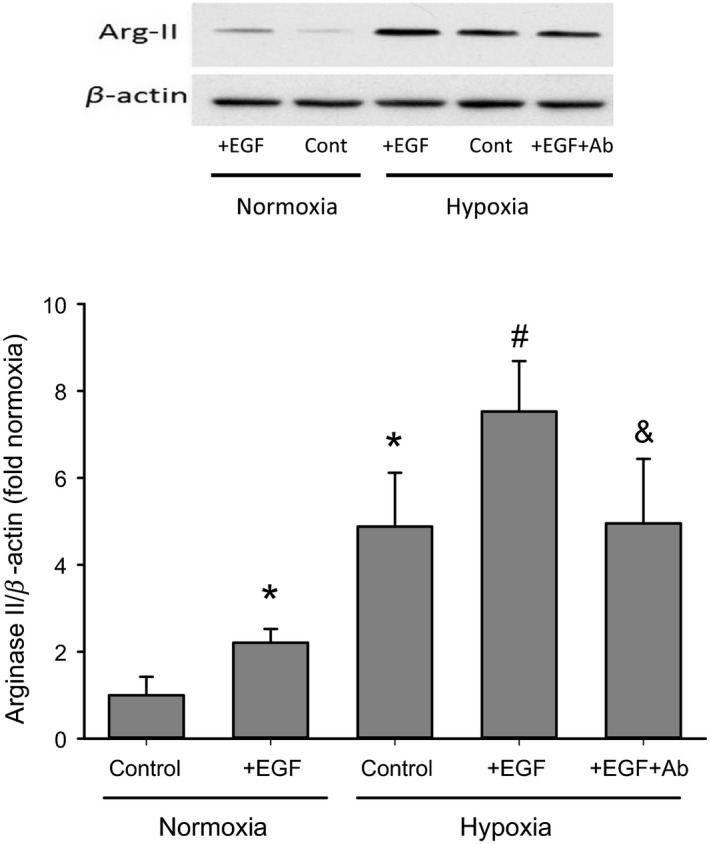
EGF increased arginase II protein expression in hPMVEC. hPMVEC were treated with vehicle (control) or 50 ng/mL epidermal growth factor (+EGF) added to the media and were placed in either normoxia or hypoxia for 24 h, some hypoxic EGF treated cells also had 1.0 *μ*g/mL EGF neutralizing antibody added to the media (+EGF+Ab) to determine if the EGF neutralizing antibody would reverse the changes caused by the addition of exogenous EGF; *n* = 6 for each group. Typical Western blot for arginase II and *β*‐actin is shown on the top and the bottom is the densitometry data shown as fold change from normoxia control. * different from normoxia control, *P* < 0.05; # hypoxia+EGF different from hypoxia control, *P* < 0.05; & hypoxia+EGF+Ab different from hypoxia+EGF, *P* < 0.05.

Given the role of EGFR activation in hypoxia‐induced arginase II expression and the substantial increase in EGF mRNA expression in hypoxic hPMVEC, we examined the role of EGF in hypoxia‐induced arginase II expression using exogenous EGF. hPMVEC were treated with either vehicle or 50 ng/mL EGF and incubated for 24 h in either normoxia or hypoxia. An additional group of hPMVEC were treated with both 50 ng/mL EGF and 1.0 *μ*g/mL EGF neutralizing antibody and incubated in hypoxia for 24 h. The EGF neutralizing antibody prevents EGF interactions with surface receptors by binding EGF. The protein from the hPMVEC was isolated for immunoblotting for arginase II and *β*‐actin. In hPMVEC incubated in normoxia, addition of EGF to the media increased arginase II protein levels by ~twofold compared to normoxic vehicle‐treated hPMVEC (Fig. 3). Similarly, the addition of EGF to the media of hPMVEC incubated in hypoxia resulted in greater arginase II protein levels than in hPMVEC treated with vehicle in hypoxia (Fig. 3). Treatment of hypoxic hPMVEC with the EGF and the EGF neutralizing antibody resulted in significantly lower arginase II protein levels than in hypoxic hPMVEC treated with EGF alone, demonstrating that the EGF neutralizing antibody does block EGF effects and in this case blocked nearly all of the effect of exogenously added EGF (Fig. 3).

**Figure 4:**
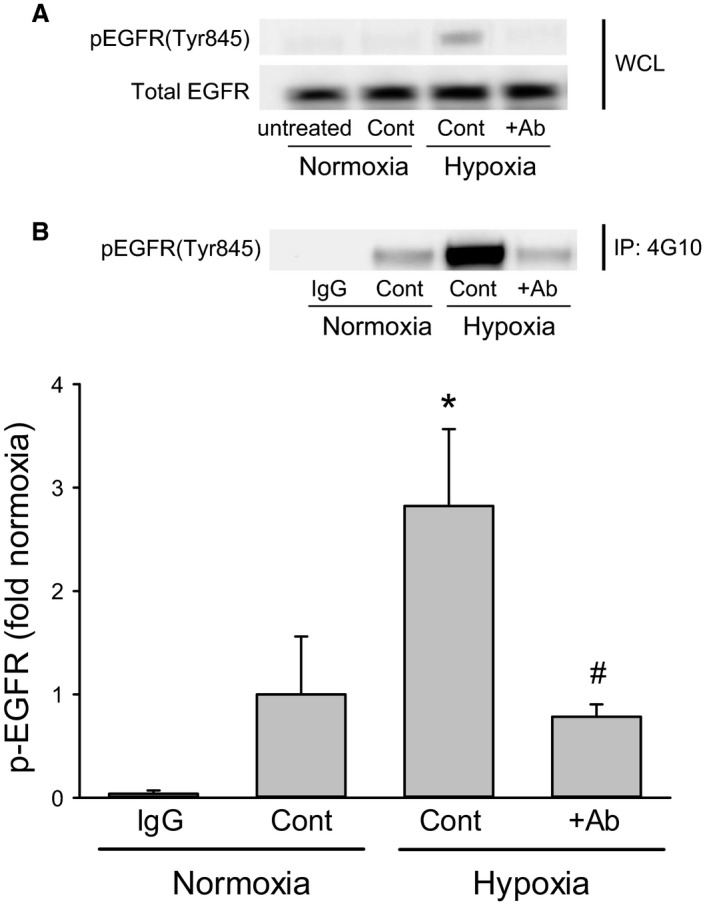
Hypoxia increased phosphorylated EGFR which was prevented by the EGF neutralizing antibody. hPMVEC were not treated or treated with vehicle (cont) and placed in normoxia for 24 h (untreated), or treated with vehicle (cont) or with an EGF neutralizing antibody (+EGF Ab) and placed in hypoxia for 24 h. The protein was harvested. (A) Some of the protein (WCL, whole‐cell lysate) was used for Western blotting for pEGFR and total EGFR and representative Western blots are shown. (B) The remainder of the protein was used for immunoprecipitation (IP), where 4G10 (anti‐pTyr antibody) was used for pull down with IgG used as a negative control for pull down. The immunoprecipitate was used for Western blotting with an antibody against pEGFR(Tyr845). A representative Western blot of immunoprecipitate showing that hypoxia (control) resulted in increased pEGFR(Tyr845), which was prevented by the EGF Ab (+Ab). Densitometry from three experiments shown as fold change from normoxia control (note the IgG negative pull down control is an *n* of 2). * hypoxia control different from normoxic control, *P* < 0.05; # hypoxic + Ab different from hypoxic control, *P* < 0.05.

To determine if hypoxia resulted in EGF‐mediated activation of EGFR, we utilized immunoprecipitation with 4G10 (anti‐phosphotyrosine antibody) and Western blotting with an antibody against phospho‐EGFR (pEGFRTyr845). hPMVEC were not treated or treated with vehicle and placed in normoxia for 24 h; treated with vehicle or with an EGF neutralizing antibody (+EGF Ab; 1.0 *μ*g/mL) and placed in hypoxia for 24 h. The protein was harvested and used for immunoprecipitation. Hypoxia (control) resulted in a nearly threefold greater levels of pEGFR(Tyr845) than the levels found in normoxic control cells (Fig. 4). When hPMVEC were incubated in hypoxia with the EGF Ab added to the media, the levels of pEGFR(Tyr845) were significantly lower than in hypoxia control hPMVEC and not different from normoxic controls (Fig. 4).

**Figure 5:**
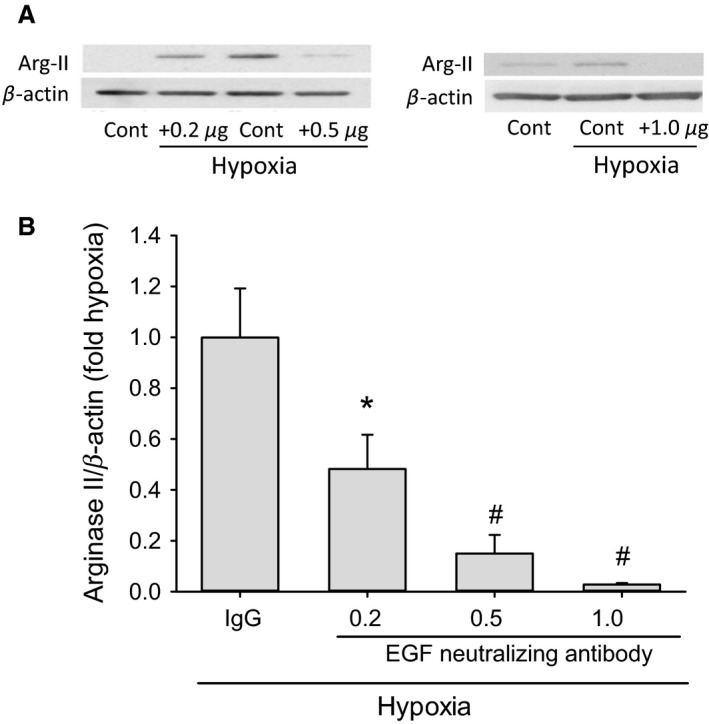
Preventing EGF interactions with surface receptors using the EGF neutralizing antibody prevented hypoxia‐induced arginase II protein expression. hPMVEC were treated with either IgG or three different doses of the EGF neutralizing antibody (0.2, 0.5, or 1.0 *μ*g/mL) and then incubated in hypoxia for 24 h; *n* = 5 for each group except the 1.0 *μ*g/mL dose where *n* = 3. (A) Typical Western blots for arginase II and *β*‐actin are shown. (B) The densitometry data shown as fold change from hypoxia + IgG (control). * different from IgG, *P* < 0.05; # different from IgG, *P* < 0.005.

To further examine the effect of preventing EGF interactions with surface receptors like EGFR on hypoxia‐induced arginase II expression in hPMVEC, we utilized varying doses of the EGF neutralizing antibody. The cells were treated with either IgG or three different doses of the EGF neutralizing antibody (0.2, 0.5, or 1.0 *μ*g/mL) and then incubated in hypoxia for 24 h. One group of isotype IgG treated hPMVEC were incubated in normoxia (Fig. 5B). Treatment with the EGF neutralizing antibody significantly attenuated hypoxia‐induced arginase II protein levels, with the 1.0 *μ*g/mL dose completely preventing hypoxia‐induced arginase II protein levels (Fig. 5B).

### Treatment with the EGF neutralizing antibody significantly reduced hypoxia‐induced proliferation of hPMVEC

**Figure 6:**
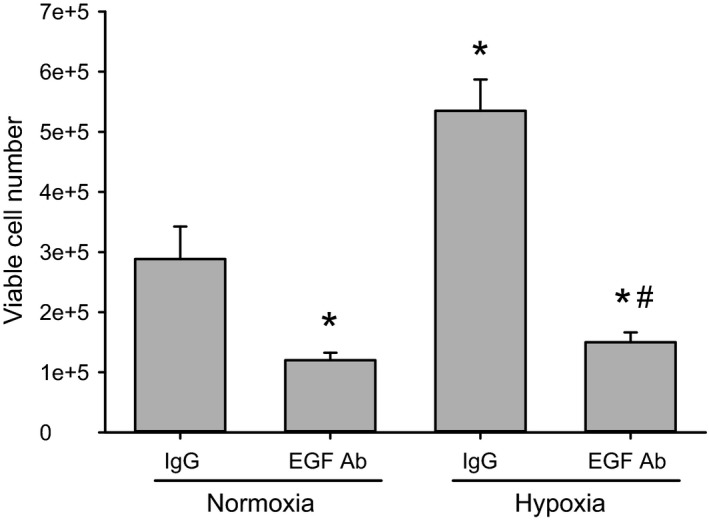
Treatment with the EGF neutralizing antibody significantly reduced hypoxia‐induced proliferation of hPMVEC. The same number of hPMVEC (5 × 10^4^) were plated in each well of a six‐well plate, the hPMVEC treated with IgG (0.5 *μ*g/mL) or EGF neutralizing antibody (0.5 *μ*g/mL), and then placed in either normoxia or hypoxia for 48 h. For each group *n* = 3. After 48 h, the numbers of viable cells were counted using trypan blue exclusion. * different from IgG normoxia, *P* < 0.05; # EGF Ab hypoxia different from IgG hypoxia, *P* < 0.001.

To determine the role of EGF interactions with surface receptors on hypoxia‐induced hPMVEC proliferation, we again used the EGF neutralizing antibody. The same number of hPMVEC (5 × 10^4^) were plated in each well of a six‐well plate, the hPMVEC were treated with isotype IgG (0.5 *μ*g/mL) or EGF neutralizing antibody (0.5 *μ*g/mL), and then placed in either normoxia or hypoxia for 48 h. After 48 h, the numbers of viable cells were counted using trypan blue exclusion. The normoxic hPMVEC treated with the EGF neutralizing antibody had fewer viable cells than did the normoxic hPMVEC treated with isotype IgG control (Fig. 6). Treatment of hypoxic hPMVEC with the EGF neutralizing antibody resulted in viable cell numbers similar to those seen with normoxic EGF neutralizing antibody‐treated hPMVEC and substantially fewer viable cells than in the hypoxic hPMVEC treated with isotype IgG control (Fig. 6).

### Preventing hypoxia‐induced EGF expression inhibited hypoxia‐induced arginase II protein expression and the hypoxia‐induced increase in viable cell numbers

**Figure 7:**
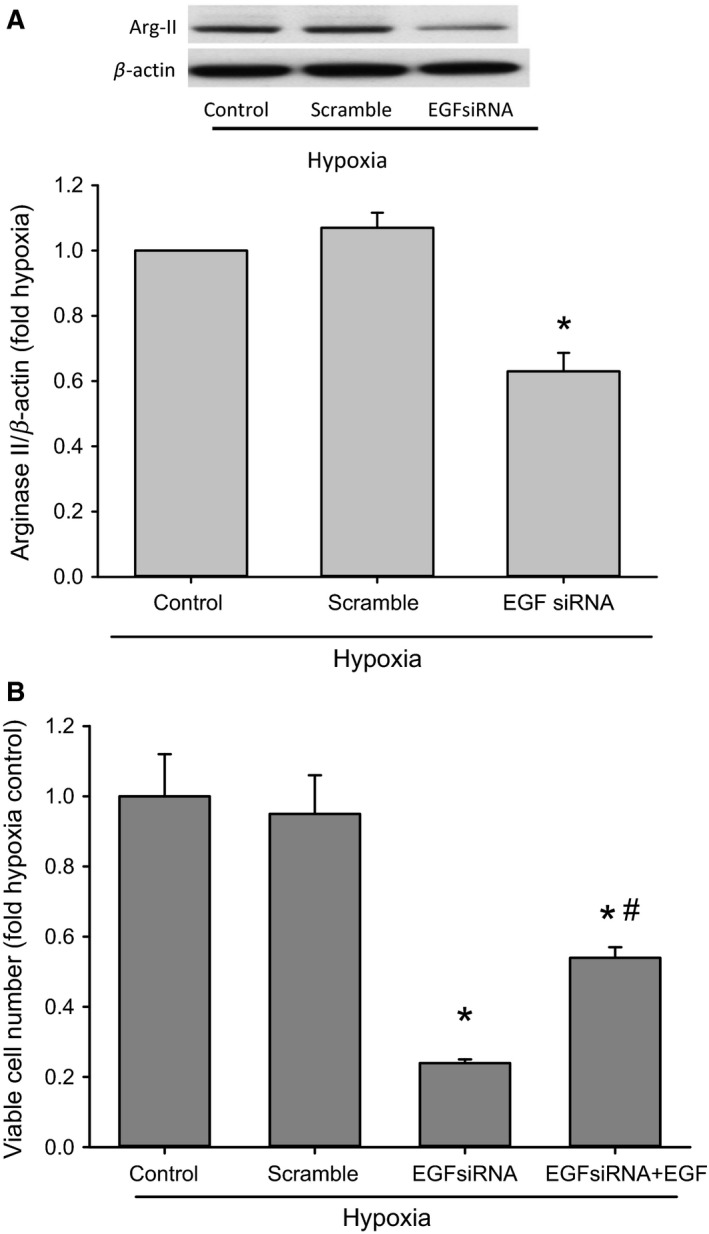
Preventing hypoxia‐induced EGF expression inhibited hypoxia‐induced arginase II protein expression and the hypoxia‐induced increase in viable cell numbers. (A) hPMVEC were treated with scramble siRNA or EGF siRNA for 24 h. Non‐transfected hPMVEC were also utilized as controls. The hPMVEC were then washed and allowed to recover in normoxia for 48 h before being incubated in hypoxia for 24 h. Protein was isolated for immunoblotting for arginase II; *n* = 7 for each group. A typical Western blot for arginase II and *β*‐actin are shown on top and the densitometry data for arginase II normalized to *β*‐actin are shown below as fold change from hypoxia control. * hypoxia EGF siRNA different from both hypoxia control and hypoxia scramble, *P* < 0.05. (B**)** hPMVEC were treated with scramble siRNA or EGF siRNA using transfection reagent for 24 h as above in A, and nontransfected hPMVEC were also utilized as controls. hPMVEC were then washed and allowed to recover in normoxia for 24 h, cells were then trypanized and resuspended and 5 × 10^4^ hPMVEC were plated in each well of a six‐well plate and placed in hypoxia for 48 h. An additional group of hypoxic hPMVEC transfected with EGF siRNA had 50 ng/mL EGF added to the media. After 48 h, the numbers of viable cells were counted using trypan blue exclusion. Each group had *n* = 3. * EGF siRNA different from control or scramble, *P* < 0.05. EGF siRNA + EGF different from EGF siRNA, *P* < 0.05.

To investigate the role of EGF expression on hypoxia‐induced arginase II expression in hPMVEC, we utilized siRNA techniques. hPMVEC were transfected with scramble siRNA or EGF siRNA for 24 h. Non‐transfected hPMVEC were also utilized as controls. The hPMVEC were then washed and allowed to recover in normoxia for 48 h before being placed in hypoxia for 24 h. Protein was isolated for immunoblotting for arginase II. There was no significant difference in arginase II protein levels between the nontransfected hPMVEC incubated in hypoxia and the scramble transfected hPMVEC incubated in hypoxia (Fig. 7A). However, the hypoxic hPMVEC transfected with the EGF siRNA had arginase II protein levels that were significantly lower than either nontransfected hypoxic hPMVEC or scramble transfected hypoxic hPMVEC (Fig. 7A).

To determine the role of EGF expression on hypoxia‐induced hPMVEC proliferation, we determined viable cell numbers following transfection with either scramble or EGF siRNA. hPMVEC were treated with scramble siRNA or EGF siRNA using transfection reagent for 24 h as above, and nontransfected hPMVEC were also utilized as controls. hPMVEC were then washed and allowed to recover in normoxia for 48 h, cells were then trypanized and resuspended and 5 × 10^4^ hPMVEC were plated in each well of a six‐well plate and placed in hypoxia for 48 h. A group of EGF siRNA transfected cells had 50 ng/mL EGF added to the media. After 48 h, the numbers of viable cells were counted using trypan blue exclusion. After 48 h in hypoxia, there were no differences in viable cell numbers between nontransfected hPMVEC and scramble transfected hPMVEC (Fig. 7B). On the other hand, hypoxic hPMVEC transfected with EGF siRNA had substantially fewer viable cells than did either non‐transfected hypoxic hPMVEC or scramble transfected hypoxic hPMVEC (Fig. 7B). Furthermore, the treatment of EGF siRNA transfected hypoxic hPMVEC with exogenous EGF resulted in significantly more viable cells after 48 h than in hypoxic EGF siRNA transfected hPMVEC (Fig. 7B).

### EGF released from hypoxic hPMVEC promotes an increase in both arginase II protein expression and viable cell numbers in hPASMC

**Figure 8:**
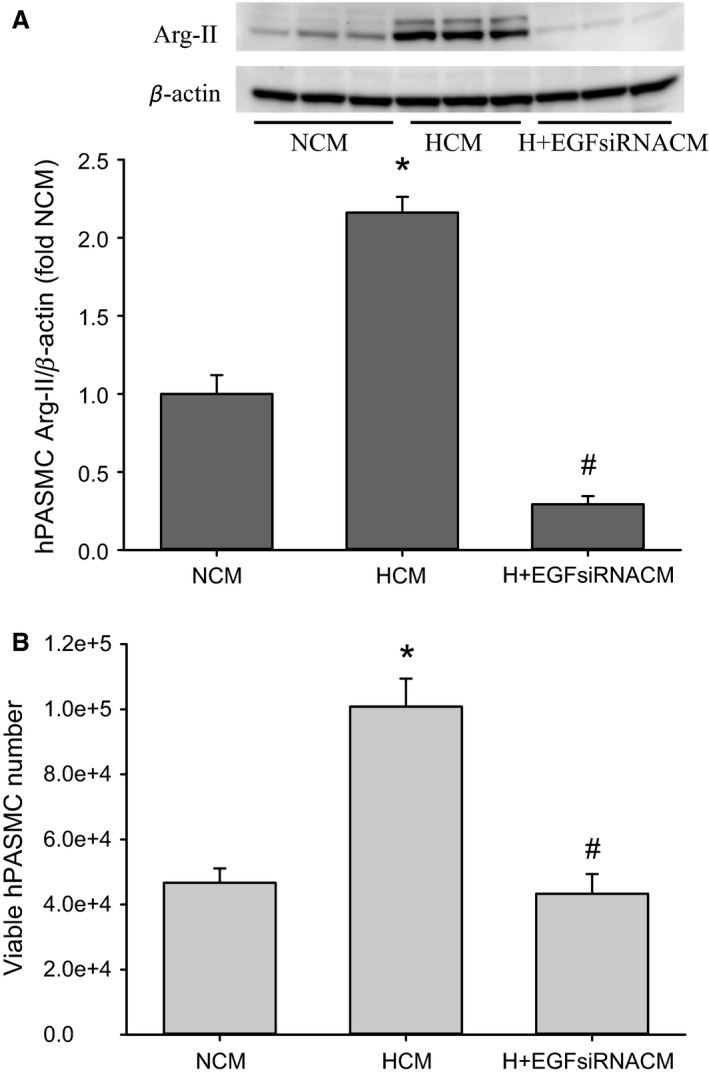
EGF released from hypoxic hPMVEC promotes an increase in arginase II expression and in viable cell numbers in hPASMC. Conditioned media were obtained from hPMVEC incubated in either normoxia (designated NCM) or hypoxia (designated HCM) for 24 h. Additionally, some hPMVEC were transfected with an EGF siRNA for 24 h, allowed to recover in fresh media for 48 h before having fresh media placed on them and incubated in hypoxia for 24 h to yield conditioned media, which we designated H+EGFsiRNACM. (A) The conditioned media from each of the three different hPMVEC groups were mixed 1:1 with SmGM and placed on hPASMC and the hPASMC were incubated for 24 h in normoxia. Protein was isolated for immunoblotting for arginase II; *n* = 3 for each group. Western blots for hPASMC arginase II and *β*‐actin are shown on top and the densitometry data for hPASMC arginase II normalized to *β*‐actin are shown below as fold change from NCM control. * HCM different from NCM, *P* < 0.001. # H+EGFsiRNACM different from HCM, *P* < 0.001. (B**)** Conditioned media from each of the three different hPMVEC groups were mixed 1:1 with SmGM and placed on equal numbers of hPASMC (*n* = 3 for NCM and H+EGFsiRNACM and *n* = 6 for HCM) in each well of six‐well plates (1 × 10^4^). The hPASMC were incubated for 120 h in normoxia and then viable cell numbers determined by trypan blue exclusion. * HCM different from NCH, *P* < 0.005; # H+EGFsiRNACM different from HCM, *P* < 0.005.

Endothelial cells can also release soluble factors that can act on other cells in the vessel wall to promote proliferation and vascular remodeling. We tested the hypothesis that EGF released from hypoxic hPMVEC would promote hPASMC proliferation as evidenced by an increase in viable hPASMC numbers. We utilized conditioned media from hPMVEC incubated for 24 h as previously described (White et al. 2017). The conditioned media harvested from hPMVEC incubated in normoxia were designated NCM, whereas the conditioned media harvested from hPMVEC incubated in hypoxia were designated HCM. Conditioned media were also obtained from hPMVEC that were transfected with EGF siRNA for 24 h, washed and fresh media placed on them for a 48‐h recovery period, and then washed, fresh media placed on them, and incubated in hypoxia for 24 h to yield the conditioned media designated H+EGFsiRNACM. In the first set of experiments examining arginase II protein levels, conditioned media from the three different hPMVEC groups were placed on hPASMC and the hPASMC were incubated for 24 h in normoxia. Protein was isolated for immunoblotting for arginase II. hPASMC incubated in normoxia with HCM had ~twofold greater arginase II protein levels than did hPASMC incubated in normoxia with NCM (Fig. 8A). However, the hPASMC incubated in normoxia with H+EGFsiRNACM had significantly lower arginase II protein levels than hPASMC incubated in normoxia with NCM (Fig. 8A). In a second set of experiments examining viable cell numbers, the conditioned media from the hPMVEC were mixed 1:1 with SmGM and placed on equal numbers of hPASMC in each well of six‐well plates. The plates were incubated for 120 h in normoxia and then viable cell numbers determined. The hPASMC incubated in NCM had an increase in cell number of ~fourfold over 120 h, whereas hPASMC incubated in HCM had an increase in cell number of ~10‐fold over 120 h (*P* < 0.005 compared to NCM) (Fig. 8B). When using the H+EGFsiRNACM the viable hPASMC numbers were significantly (*P* < 0.005) lower than in hPASMC grown with HCM, and were not different from hPASMC grown with NCM (Fig. 8B). Taken together, these results suggest that hypoxic hPMVEC released EGF into the media that stimulated an increase in hPASMC viable cell numbers.

## Discussion

The main findings of this study in hPMVEC were that (1) hypoxia led to greater EGF, EGFR, and arginase II mRNA expression; (2) hypoxia led to increased pEGFR levels; (3) inhibition of EGFR activation, either using a blocking antibody to prevent ligand‐mediated activation or a small molecule inhibitor of EGFR tyrosine kinase function, attenuated hypoxia‐induced arginase II expression; (4) EGF treatment of hPMVEC resulted in upregulation of arginase II expression through a mechanism dependent on EGF interaction with EGFR; and (5) inhibition of EGF interaction with EGFR or inhibition of EGF expression prevented the hypoxia‐induced increase in both arginase II expression and viable cell numbers. We also found that conditioned media from hypoxic hPMVEC resulted in greater hPASMC proliferation, which were dependent on EGF production in the hPMVEC. Taken together, these findings support our hypothesis that hypoxia results in enhanced secretion of EGF from hPMVEC and that hPMVEC‐secreted EGF binds to EGFR to activate the signaling cascade ultimately leading to arginase II induction and cell proliferation.

We found that in hPMVEC hypoxia led to greater EGF, EGFR, and arginase II mRNA expression. We have previously shown that hypoxia increased EGFR mRNA expression in hPMVEC (Toby et al. 2010). Furthermore, we previously demonstrated that hypoxia increased arginase II protein expression in hPMVEC (Toby et al. 2010; White et al. 2017). We also found that hypoxia increased EGFR activation through EGF binding as demonstrated by increased pEGFR protein levels which were inhibited by the EGF neutralizing antibody, a finding consistent with our previous study in hPMVEC (White et al. 2017) using an AG1478‐inhibitable tyrosine kinase activity assay. Pulmonary vascular smooth muscle proliferation, a hallmark of pulmonary hypertension, has been shown to occur at least in part due to EGF‐induced cell signaling (Schultz et al. 2006). We have previously shown that exposing HeLa cells to 1% oxygen resulted in a ~20‐fold increase in EGF mRNA and a substantial increase in pEGFR protein levels (Setty et al. 2017). Our results demonstrate that hypoxia also leads to a substantial increase in EGF expression and subsequent EGFR activation in hPMVEC. The important evidence linking increased the EGF production to the activation of EGFR in hPMVEC which comes from the experiments, wherein the inhibition of EGFR using a blocking antibody to prevent ligand‐mediated activation attenuated hypoxia‐induced arginase II expression (as shown in Fig. 2) and the immunoprecipitation data for pEGFR (as shown in Fig. 4). These finding are consistent with our previous studies (Setty et al. 2017) in HeLa cells and supports the concept of a hypoxia responsive EGF‐EGFR pathway in endothelial cells leading to arginase II expression. It has been described that endothelial release of various growth factors, including EGF, can activate epidermal cells in the lung to promote lung development. For example, endothelial‐derived EGF activating EGFR in epidermal cells has been described as a mechanism supporting alveologenesis (Ding et al. 2011). However, our data demonstrate a novel hypoxia‐induced EGF signaling pathway in which hypoxia leads to EGF production in hPMVEC, and the EGF produced then interacts with EGFR on the same or nearby endothelial cells to promote arginase II expression and cellular proliferation. Interestingly, our data from the conditioned media studies suggest that EGF released from hypoxic hPMVEC may interact with pulmonary vascular smooth muscles to promote their proliferation as well. Cross‐talk between endothelial cells and vascular smooth muscle cells is well‐known, including paracrine regulation of smooth muscle cell function by endothelial cells (Humbert et al. 2008; Sakao et al. 2009; Tang et al. 2013; Deng et al. 2015; Gao et al. 2016).

We have previously demonstrated the importance of EGFR activation in arginase II induction and cell proliferation (Toby et al. 2010; White et al. 2017). In the studies described herein we demonstrate that EGF binding to EGFR is necessary for arginase II induction and cellular proliferation. We found that neutralizing EGF prevented the phosphorylation (activation) of EGFR, the hypoxia‐induced upregulation of arginase II, and the resultant increase in viable cell numbers (as shown in Figs. 4, 5 and 6). Furthermore, when using a blocking antibody for EGFR, which binds to EGFR so that ligand (EGF) cannot bind, we found that hypoxia‐induced arginase II expression was completely prevented (as shown in Fig. 2). Similarly, inhibiting the tyrosine kinase activity of EGFR using AG1478 significantly attenuated hypoxia‐induced arginase II production consistent with our previous studies (Toby et al. 2010). Indeed we have recently shown that hypoxia activates EGFR which leads to activation of ERK, and this EGFR‐ERK activation is required for hypoxia‐induced arginase II expression (White et al. 2017). Thus, taken together our studies demonstrate that hPMVEC exposed to hypoxia have enhanced release of EGF which activates EGFR leading to ERK pathway activation and arginase II induction. Furthermore, these data strongly support the necessary role of the EGF‐EGFR‐ERK‐arginase II pathway in hypoxia‐induced hPMVEC proliferation.

The literature for a role of EGF‐EGFR signaling in PH pathogenesis has gone back and forth over the last 10–15 years. For example, Le Cras et al. (2003) found that EGFR signaling was necessary for the PH caused by TGF‐*α* overexpression in mice. Merklinger et al. (2005) found that treating rats with an EGFR inhibitor dramatically improved survival in a monocrotaline‐induced model of PH. Zhou et al. (2007)) reported that EGFR inhibition prevented platelet activating factor‐induced pulmonary vascular smooth muscle cell proliferation. On the other hand, Dahal et al. (2010) reported that although EGFR inhibitors improved PH in rats caused by monocrotaline, EGFR inhibitors had little effect on hypoxia‐induced PH in mice. Dahal et al. (2010) also reported that EGFR protein expression although higher in patients with idiopathic pulmonary arterial hypertension (IPAH) did not reach statistical significance. The findings of Dahal et al. (2010) led to the notion that perhaps EGFR signaling was not involved in the pathogenesis of PH. Around the same time, we reported that in hPMVEC hypoxia‐induced proliferation depended on EGFR activation (Toby et al. 2010). Shortly thereafter Overbeek et al. (2011) reported focal EGFR immunoreactivity in lung sections from patients with IPAH but none in lung sections from patients without IPAH. Recently, Rafikova et al. (2016) published their findings in lung sections from controls and patients with PH of no difference in EGFR protein levels, but significantly greater amounts of activated EGFR protein, as measured by dimerization and phosphorylation at tyrosine1068, than in controls. This finding is consistent with increased EGFR signaling despite no increase in EGFR protein levels. Zhou et al. (2015) recently reported that in COPD patients a SNP in the EGFR gene significantly increased the risk for developing PH. Thus, although there are some conflicting reports in the literature concerning the role of EGFR in PH, recent human studies strongly suggest that activation of EGFR is an important contributor to the development of PH. Furthermore, Kelly et al. (2017) very recently reported on a GWAS study in mice that the EGFR gene was involved in high fat diet‐induced PH.

In conclusion, we found that hypoxia induces EGF production in hPMVEC, and the hypoxia‐induced EGF activates EGFR leading to increased arginase II expression and cellular proliferation. These studies demonstrate that EGF is required for the hypoxia‐induced activation of EGFR. Taken together with findings from our previous studies demonstrate that the EGF‐EGFR‐ERK‐arginase II pathway is critical for hypoxia‐induced proliferation in hPMVEC. We speculate that EGF‐EGFR signaling may be a novel therapeutic target for pulmonary hypertensive disorders associated with hypoxia.

## Conflict of Interest

The authors have no conflicts of interest to declare.
